# A Study of the Change in Sodium and Potassium Ion Concentrations in Stored Donor Blood and Their Effect on Electrolyte Balance of Recipients

**DOI:** 10.1155/2019/8162975

**Published:** 2019-09-29

**Authors:** Samuel Antwi-Baffour, Jonathan Kofi Adjei, Felix Tsyawo, Ransford Kyeremeh, Felix Abekah Botchway, Mahmood Abdulai Seidu

**Affiliations:** ^1^Department of Medical Laboratory Sciences, School of Allied Health Sciences, College of Health Sciences, University of Ghana, P.O. Box KB 143, Korle-Bu, Accra, Ghana; ^2^Department of Haematology, Ho Municipal Hospital, Ho, Volta Region, Ghana; ^3^Department of Chemical Pathology, Central Laboratory Service, Korle-Bu Teaching Hospital, Accra, Ghana

## Abstract

**Background:**

Preserved blood cells undergo progressive structural and functional changes that may affect their function, integrity, and viability after transfusion. The impact of transfusion of stored blood on potassium, sodium, or acid-base balance in the recipient may be complex, but information on it is inconsistent. This study therefore sought to determine the changes in the potassium and sodium levels in whole blood stored at 4°C for 28 days and clinical outcomes when such blood are transfused.

**Methods:**

Whole blood were taken into double CPDA-1 bags and 50 ml transferred into the satellite bags for the study. Electrolyte concentration determinations were made on each of the blood sample on days 0, 7, 14, 21, and 28 using the Vitalab Selectra Junior chemistry analyser. The remaining blood in the main bags was transfused after the 28-day period, and biochemical analysis carried out on the patients before and after the transfusion. One-way ANOVA was used for the analysis of variance between the weekly ion concentrations and independent sample Mann–Whitney *U* test for the data obtained from the patients.

**Results:**

The mean potassium level of all the samples started with a normal value of 3.45 mmol/L on the first day followed by a sharp rise to 9.40 mmol/L on day 7, 13.40 mmol/L on day 14, 14.60 mmol/L on day 21, and 15.40 mmol/L on day 28. Sodium on the other hand started with a high value of 148.4 mmol/L on day 0 and then reduced to 146.4 mmol/L on day 7, 140.8 mmol/L on day 14, 135.6 mmol/L on day 21, and a low value of 130.8 mmol/L on day 28. No adverse clinical outcomes were seen in patients after they were transfused with the blood.

**Conclusion:**

It can be deduced that potassium concentration in refrigerated blood increases, whilst sodium concentration reduces with time and when such blood is transfused, it may not result in any adverse clinical outcome.

## 1. Introduction

Blood transfusion is a vital life-saving measure and is administered under various pathological conditions. While blood component therapy has become the standard practice in the developed world, millions of whole blood are transfused annually in resource limited countries bringing into question the aspect of long-term storage and preservation [1]. The Food and Drug Administration (FDA) of the USA has set storage period of up to 35 days for blood anticoagulated in citrate phosphate dextrose adenine-1 (CPDA-1) which has been accepted in many countries worldwide [2]. It has however been postulated that when blood for transfusion has been stored for so long, it increases the risk of transfusion complications. This is because red blood cells (RBCs) undergo both structural and functional changes which can affect their posttransfusion overall viability and function [2].

Furthermore, the bioreactive substances such as histamine, lipids, and cytokines released by the “passenger” leucocytes that may exert direct effect on metabolic and physical changes associated with the senescence in cells are related to RBC storage medium lesion [3]. Other evidence also suggests that hypothermic storage of red blood cells may lead to reduced metabolism and energy demand, subsequently rendering ATP-dependent sodium potassium pump inoperative and ultimately leading to the free movement of sodium into the cells and potassium out of the cells [4]. Current research indicates that RBC hypothermic storage lesion is responsible for the association of blood transfusion with an increased length of stay in the hospital, increased infections, multiple organ system failure, and ultimately increased morbidity and mortality [5].

Hyperkalaemia is defined as a serum potassium level above the reference range, and arbitrary thresholds such as >5.00, >5.50, or >6.00 mmol/L are used to indicate degree of severity [6]. In situations where some comorbid conditions and other factors that interfere with the excretion of kidney potassium are seen, patients with chronic and advanced kidney diseases may be at high risk of increased plasma potassium [6]. It has also been seen that increased plasma potassium tend to be high in people with chronic kidney disease than in the general population [6]. This is because the kidneys play a major role in maintaining potassium homeostasis by matching potassium intake with potassium excretion. Any increase in serum potassium levels in people with severe hyperkalaemia especially the critically ill cases may result in serious complications and even death [7].

The major electrolyte in the extracellular fluid (ECF) is sodium which has about 98% of its total quantity in the ECF, and only about 2% is found in the intracellular fluid (ICF). Sodium has a reference range of 135.0–145.0 mmol/L. Subsequently, sodium levels above 145.0 mmol/L will result in hypernatremia, which is generally associated with a hyperosmolar state. When extracellular sodium is increased, it causes intracellular fluid to escape out of cells into extracellular spaces and this may result in cellular dehydration. On the other hand, a sodium level below 135.0 mmol/L is low and may result in a hyponatraemia condition [8]. This can cause cellular oedema which may affect the central nervous system as well as depression and cerebral oedema [9].

The impact of transfusion of stored blood on the potassium or sodium and acid-base balance in the recipient is very complex. It is, however, largely dependent on the volume of blood that is transfused, the rate of transfusion, the rate of citrate metabolism, and the changing state of the peripheral perfusion of the patient/recipient [10]. Failure to establish the apparent electrolyte changes has been found to be fatal in some instances [10]. This study therefore sought to determine the changes in the potassium and sodium levels in whole blood stored at 4°C and over 28-day period in order to eliminate a potential source of high potassium vs. low sodium for those with severe hyperkalaemia vs. hyponatremia requiring blood transfusion in resource-limited settings.

## 2. Materials and Methods

### 2.1. Ethics

Ethical approval was sought from the Ethics and Protocol Review Committee of the School of Biomedical and Allied Health Sciences before the study was carried out. Written informed consent was obtained from the blood donors and recipients before the study commenced. Written informed consent was also sought from the blood bank and the management of the Ho municipal hospital.

### 2.2. Procedure

#### 2.2.1. Sampling Method

Thirty donated whole blood that have been screened using the protocol of the transfusion medicine unit of the hospital were used for the study. Approximately 450 ml of whole blood were taken into double citrate phosphate dextrose adenine-1 (CPDA-1) bags containing 63 ml of the anticoagulant bringing the total volume to approximately 513 ml. Each bag therefore had an average blood volume of about 500 ml. Following this, 50 ml of the thoroughly mixed blood were transferred into the satellite bags and stored at 4°C to be used for the study. The remaining blood (≈450 ml) in the main bags were transfused to 16 patients (3 received 3 units each, 8 received 2 units each, and 5 received 1 unit each) after the 28-day period. The transfusion took between 2 and 3 hours depending on the patient's condition to complete. Five electrolyte concentration determinations were made on each of the 30 samples on day 0 (before storage), day 7, day 14, day 21, and day 28 of storage. Venous samples were taken from patients on the first day of transfusion, second and third days, and the average of the values obtained was used to compute the posttransfusion biochemical markers. On the respective days of analysis, 2 ml of the whole blood sample was placed into a gel separator tube and spun at 1500 rpm for 3 minutes to obtain plasma which was then analysed for sodium, potassium, and chloride using the ion-selective electrode of the Vitalab Selectra Junior chemistry analyser (Elitech Group, Netherland). Urea and creatinine were assayed using the ELITech chemistry reagents kit from ELITech Group Clinical Systems (Paris, France). The chloride, urea, and creatinine were assayed to rule out any undiagnosed kidney disease.

#### 2.2.2. Data Analysis

The mean sodium and potassium ion concentrations for the weekly readings were determined from the stored blood and also the patient samples. The change in ion concentrations in reference to the baseline measurements was also determined. A paired *t* test was used to compare means of quantitative variables such as the relationship between the baseline sodium and potassium concentrations and the subsequent weekly measurements. The one-way ANOVA was used for analysis of variance between the weekly ion concentrations. The data from patients were expressed as median (Q1–Q3) and analysed using the independent sample Mann–Whitney *U* test.

## 3. Results

**Figure 1:**
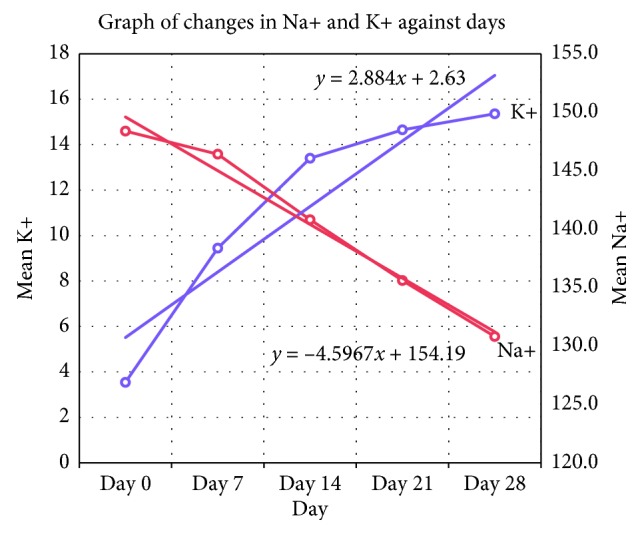
Change in potassium (K+) and sodium (Na+) concentrations in donor blood over 28 days of storage.

The mean plasma potassium level of all the samples in relation to the various days' readings started with a normal average value of 3.45 mmol/L on the first day (day 0), followed by a sharp rise to 9.40 mmol/L up to day 7, followed by a similar increase to 13.40 mmol/L on day 14. The mean plasma potassium values however stabilised with slight increases from 14.60 mmol/L on day 21 to 15.40 mmol/L on day 28. The mean plasma sodium on the other hand started with a high value of 148.4 mmol/L on the first day and then reduced to 146.4 mmol/L on day 7, 140.8 mmol/L on day 14, 135.6 mmol/L on day 21, and a low value of 130.8 mmol/L on day 28 (Figure 1).

The trend line from the graph indicates that, with initial mean plasma potassium concentration of 2.63 mmol/L, there was a unit rise in plasma concentration of 2.88 mmol/L per week (7 days) whilst with an initial mean concentration of 154.19 mmol/L, plasma sodium decreased by 4.57 mmol/L per week (7 days). This means that there was a steady increase of plasma potassium and a steady decrease in plasma sodium as the whole blood aged in storage. The rate of increase of plasma potassium however slowed down with aging erythrocytes while the rate of decrease of plasma sodium remained the same from day 7 onward.

Table 1 shows the mean and standard deviation of potassium and sodium on each of the days examined.

The maximum change in potassium concentration over the storage period was 170.00% increase in the basal value of 3.45 mmol/L recorded in the first seven days. The increase however slowed down after day 21. In all, there was a total increase of 11.9 mmol/L (224.42%) from the day of blood donation to the 28^th^ day. The total change in the sodium between the start to the end of the study was 17.6 mmol/L (12.38%). The changes in concentrations of the analytes are presented in Table 2.

The one-way ANOVA was used for analysis of variance between the weekly ion concentrations (Table 3). As can be seen, the variation between groups and within groups for both analytes was significant.

A follow-up study was carried out where the units of blood under investigation were transfused at the end of the storage period and the patients monitored for clinical effects. The patients who received the transfusion were postoperative surgical patients with haemoglobin ≤8.0 g/dl. There was pretransfusion analysis of the analytes of interest before the posttransfusion analysis was carried out (Table 4).

## 4. Discussion

Blood transfusion as a therapeutic procedure can be harmful instead of saving lives, and this is because every transfusion is carried out with it a potential risk for the recipient [11]. The transfusion of whole blood with relatively high potassium concentration and low sodium concentration has been associated with worse outcomes in several populations of patients, including critically ill patients [12]. The blood transfusion services which have a duty of care towards blood recipients must therefore take steps to forestall these occurrences. Determining what happens during storage of blood with regards to sodium and potassium is therefore in line with such efforts. This study was therefore carried out to determine the changes in potassium and sodium ion concentrations in stored donor blood over a 28-day storage period and their effects on the electrolyte balance in recipients when such blood are transfused.

The results showed a general steady increase of plasma potassium with a steady decrease in plasma sodium over the 28-day storage period as the whole blood aged in the storage. The readings started with a normal average potassium value of 3.45 mmol/L on the first day which was followed by a sharp rise in value (9.40 mmol/L) on day 7. This was followed by a similar increase in value to 13.40 mmol/L on day 14. There values however stabilised with only slight increase to 14.60 mmol/L on day 21 and 15.40 mmol/L on day 28. The results of plasma sodium on the other hand started with a high value of 148.4 mmol/L on the first day and then reduced to 146.4 mmol/L on the 7^th^ day. This was followed by a decrease to 140.8 mmol/L on day 14, 135.6 mmol/L on day 21, and a low value of 130.8 mmol/L on the 28^th^ day. The rate of increase of plasma potassium however slowed down with aging whole blood while the rate of decrease of plasma sodium remained the same from day 7 onward. The trend line from Figure 1 indicates that with an initial mean plasma potassium concentration of 2.63 mmol/L, there was a unit rise in plasma concentration of 2.88 mmol/L per week whilst sodium had an initial mean concentration of 154.2 mmol/L that decreased by 4.6 mmol/L per week.

At the end of the 28 days, the units of stored blood were transfused and biochemical components of the patients examined to see if any moderation has taken place. Even though we observed a marginal increase in all analytes except sodium when basal values were compared with posttransfusion values, no significant changes were seen (Table 4). This finding supports the assertion that red blood cells can be stored for a period of 35 days or more, without much clinical adverse outcomes in patients. The chloride, serum urea, and creatinine in the blood of the transfused patients were estimated as a way of finding out if any of them had any renal impairment that could have bearings on the results of the potassium and sodium. It was realised that all three analytes showed marginal increases between the pre- and posttransfusion test results but the changes were not significant. Indicators of clinical outcome of the patients included length of hospital stay, mortality, loss of pallor, increased haemoglobin levels, and transfusion reaction or complications.

The results of the study agree with a similar study by Adias et al., which also recorded an increase in potassium concentration and decrease in sodium concentration during storage of blood [13]. Furthermore, the overall daily changes in both potassium and sodium agree with the work done by others that showed that the plasma level of potassium may increase by 0.5–1.0 mmol/L per day of refrigeration [14]. Other research works have also indicated that RBCs restore intracellular potassium by active transport after the transfused RBCs recover metabolic activity and adenosine-5′-triphosphate production [15]. However, paediatric patients and those with renal failure may not be able to handle this potassium load effectively and could lead to complication and even death if transfused with blood stored for longer periods. The lack of expected adverse clinical outcome might have been due to the insufficient blood units transfused which is a limitation in the study. Other limitations worthy of mention are the sample size that perhaps can be increased and the storage period that can be extended the in future work.

## 5. Conclusion

From the results of the study, it can be deduced that plasma potassium concentration in refrigerated blood increases with storage time whilst plasma sodium concentration decreases with storage time. However, the rate of increase and decrease may differ, and when this blood was transfused, no clinical adverse outcomes were seen in the patients. This work has added to other published works related to blood storage, and it is our belief that the results will go a long way to inform practitioners what goes on in stored blood and who can be given what blood as far as blood transfusion therapy is concerned as well as help address some of the storage issues associated with blood transfusion.

## Figures and Tables

**Table 1:** Mean and standard deviation of sodium and potassium concentrations on different days.

	*N*	Mean	Std. deviation
	Statistic	Statistic	Statistic
*Day 0*			
Na	30	148.383	1.010
K	30	3.543	0.157

*Day 7*			
Na	30	146.410	0.907
K	30	9.447	0.335

*Day 14*			
Na	30	140.803	0.516
K	30	13.403	0.354

*Day 21*			
Na	30	135.597	0.501
K	30	14.653	0.294

*Day 28*			
Na	30	130.807	0.451
K	30	15.360	0.352

**Table 2:** Changes and percentage changes in plasma potassium and sodium concentration over 28 days of storage.

Day of measurement	Potassium change in relation to the previous measurement (mmol/L)	Percentage change (%)	Sodium change in relation to the previous measurement (mmol/L)	Percentage change (%)
Day 1	—	—	—	—
Day 7	5.90	170.00	2.0	1.34
Day 14	4.00	40.00	5.6	3.82
Day 21	1.20	8.95	5.2	3.69
Day 28	0.80	5.47	4.8	3.53
Total	11.90	224.42	17.6	12.38

**Table 3:** ANOVA analysis.

		Sum of squares	df	Mean square	*F*	Sig.
Na+	Between groups	6453.613	4	1613.403	3146.657	0.000
	Within groups	74.347	145	0.513		
	Total	6527.960	149			

K+	Between groups	2872.543	4	718.136	7598.119	0.000
	Within groups	13.705	145	0.095		
	Total	2886.248	149			

**Table 4:** A table of clinical data obtained before and after transfusion of blood stored up to 28 days.

Type of analytes	Before	After	*p* value
	Median (Q1–Q3)	Median (Q1–Q3)	
Na^+^ (mmol/L)	142.0 (138–144)	141.5 (138.0–144.0)	0.973
K^+^ (mmol/L)	4.30 (4.00–4.78)	4.50 (3.93–4.80)	0.42
Cl^–^ (mmol/L)	102 (100–105)	103.5 (100–107.0)	0.259
UREA (mmol/L)	3.6 (2.4–6.88)	4.1 (2.63–6.98)	0.492
CREAT (*μ*mol/L)	77.5 (57.25–147.75)	81.5 (64.25–156.75)	0.338

*p* < 0.05 was taken as significant.

## Data Availability

The data used to support the findings of this study are available from the corresponding author upon request.
